# Supplementary material for: CoCl2 ‐triggered pseudohypoxic stress induces proteasomal degradation of SIRT4 via polyubiquitination of lysines K78 and K299
Source: FEBS Open Bio. 2023 Oct 12;13(12):2187–99. doi: 10.1002/2211-5463.13715 (PMC10699113; doi:10.1002/2211-5463.13715)
Supplement: Supplementary file 1 — Fig. S1. shRNA‐mediated depletion of SIRT4 in HEK293 cells. Fig. S2. CoCl2 treatment of HEK293‐eGFP cells does not lead to downregulation of eGFP levels. Fig. S3. Stabilization of SIRT4(H161Y) and SIRT4(DN28) mutants by treatment with the proteasome inhibitor MG132. Fig. S4. Flow cytometry‐based analysis of expression of SIRT4 and SIRT4 mutants. [file FEB4-13-2187-s001.pdf]

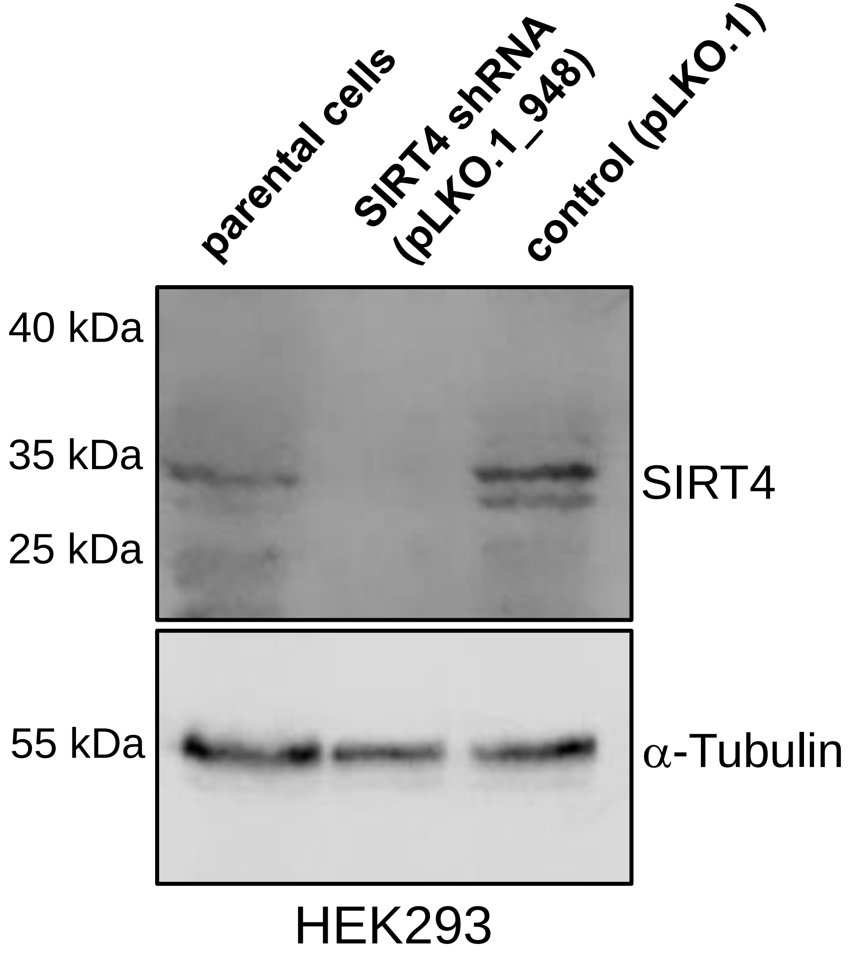

Figure S1. shRNA-mediated depletion of SIRT4 in HEK293 cells. HEK293 cell lines were generated which either express the control pLKO.1 vector or pLKO.1_948 that targets the human SIRT4 mRNA. Parental and transgenic cell lines were subjected to immunoblot analysis using a SIRT4 specific antibody.

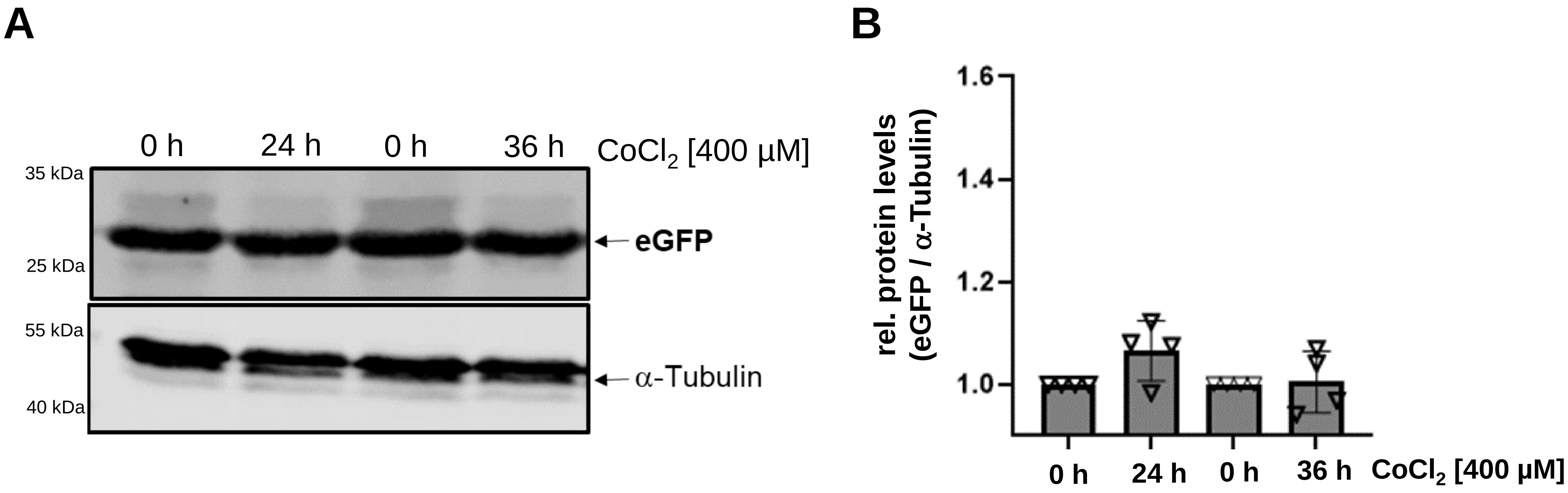

Figure S2. $CoCl_2$ treatment of HEK293-eGFP cells does not lead to downregulation of eGFP levels. (**A**) HEK293 cells stably expressing eGFP were subjected to $CoCl_2$ treatment for 24 h and 36 h followed by immunoblot analysis. (**B**) Relative quantification of eGFP immunoblot signals was performed using ImageJ based densitometric evaluation and α-Tubulin levels as loading control. To test statistical significances, a One-Way ANOVA test followed by Tukey´s Test was employed (n=4; mean ± S.D.).

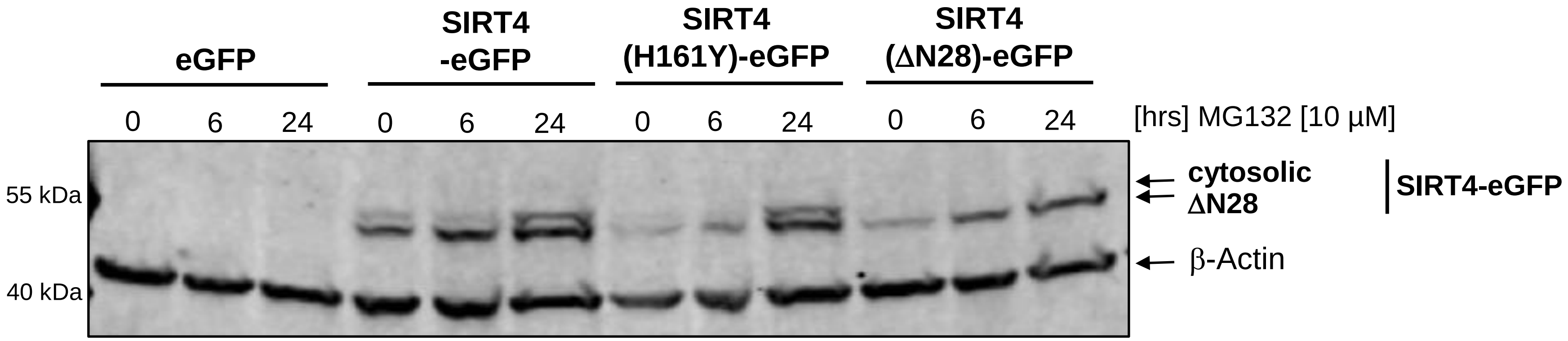

Figure S3. Stabilization of SIRT4(H161Y) and SIRT4(ΔN28) mutants by treatment with the proteasome inhibitor MG132. HEK293 cell lines expressing eGFP, SIRT4-eGFP, the catalytically inactive mutant H161Y, or the N-terminal deletion mutant SIRT4(ΔN28) that does not translocate into mitochondria, were subjected to MG132 treatment for 6 h and 24 h followed by immunoblot analysis of the respective SIRT4-eGFP/mutant SIRT4-eGFP levels. β-Actin staining served as loading control.

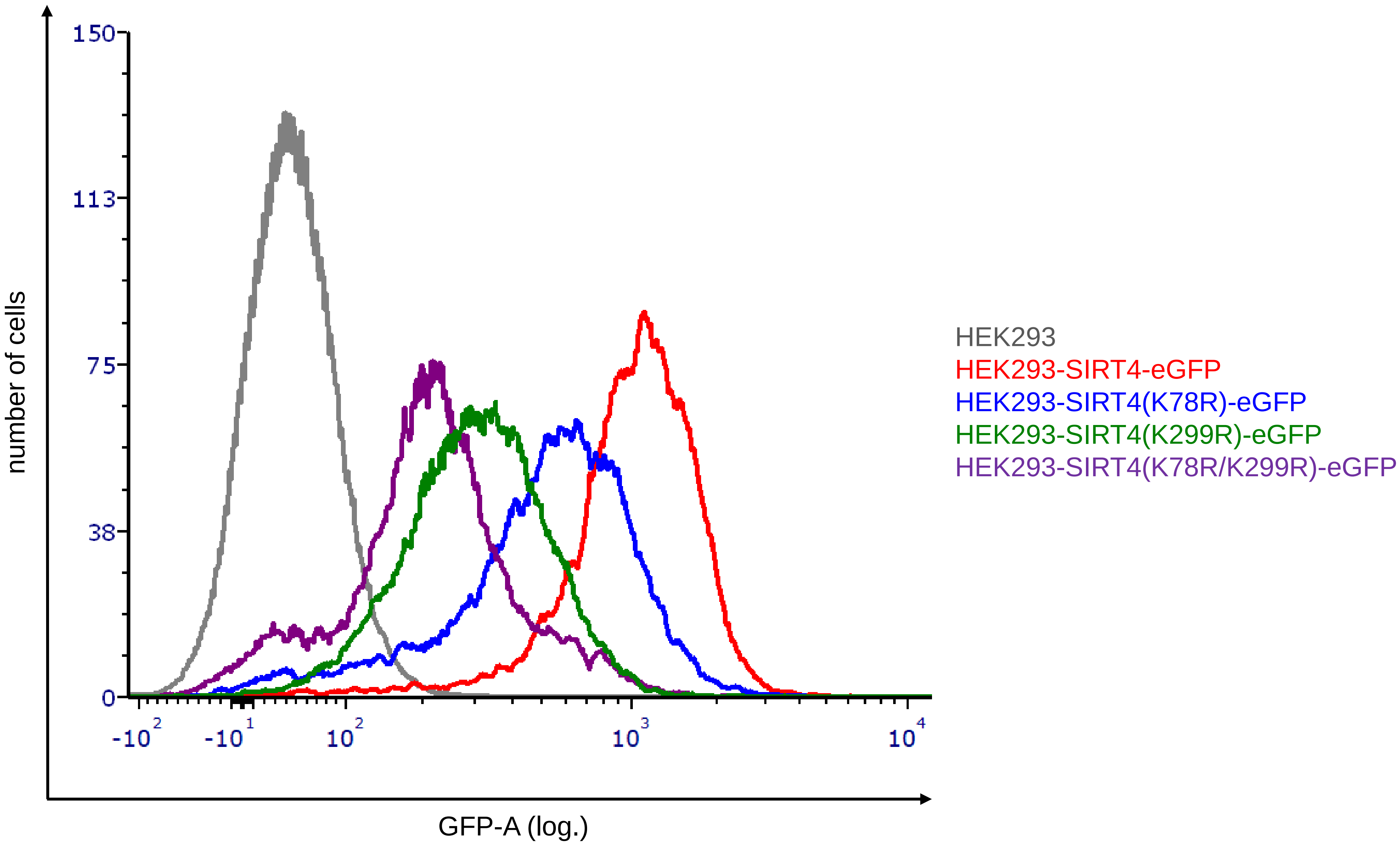

Figure S4. Flow cytometry-based analysis of expression of SIRT4 and SIRT4 mutants. Parental HEK293 cells as negative control and HEK293 cell lines stably expressing SIRT4-eGFP or SIRT4 mutants thereof were subjected to flow cytometry-based (GFP-A) expression analysis.
